# Reply to Sun et al., “Identifying Composition Novelty in Microbiome Studies: Improvement of Prediction Accuracy”

**DOI:** 10.1128/mBio.01234-19

**Published:** 2019-08-06

**Authors:** Xiaoquan Su, Gongchao Jing, Daniel McDonald, Honglei Wang, Zengbin Wang, Antonio Gonzalez, Zheng Sun, Shi Huang, Jose Navas, Rob Knight, Jian Xu

**Affiliations:** a Single-Cell Center, CAS Key Laboratory of Biofuels and Shandong Key Laboratory of Energy Genetics, Qingdao Institute of BioEnergy and Bioprocess Technology, Chinese Academy of Sciences, Qingdao, Shandong, China; b Department of Pediatrics, University of California San Diego, La Jolla, California, USA; c Department of Computer Science & Engineering, University of California San Diego, La Jolla, California, USA; d Department of Bioengineering, University of California San Diego, La Jolla, California, USA; e Center for Microbiome Innovation, University of California San Diego, La Jolla, California, USA; f Laboratory for Marine Biology and Biotechnology, Qingdao National Laboratory for Marine Science and Technology, Qingdao, Shandong, China; g University of the Chinese Academy of Sciences, Beijing, China; George Washington University; Northern Arizona University

**Keywords:** bioinformatics, community similarity, data mining, database search, microbial ecology, microbiome, microbiome novelty, novelty, search

## REPLY

To quantitatively measure the beta diversities between microbiomes, Microbiome Search Engine (MSE) (1) calculates phylogeny similarity using operational taxonomy unit (OTU) profiles; for both query and database samples, all 16S rRNA gene sequences are mapped to the Greengenes database (version 13-8) (2) for reference-based OTU picking with a 97% cutoff. Thus, in MSE, the comparison between query and database samples is approximately at the species level (3), although the actual taxonomic resolution varies according to taxon, due to differences in the evolutionary rates of the 16S rRNAs. Moreover, in MSE, both the relative abundance (with 16S rRNA gene copy number normalization [4]) and the phylogenetic structures of OTUs are utilized for similarity calculation (as in UniFrac [5, 6]), yet the speed is optimized by nonrecursive computing to enable real-time responses (7).

**FIG 1:**
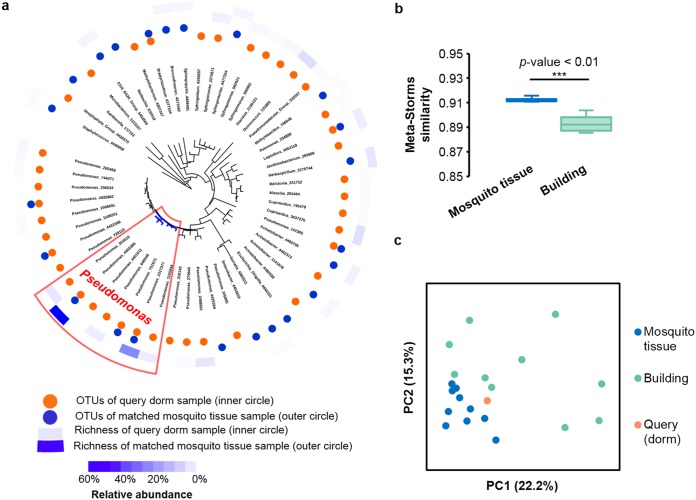
Comparison between the query microbiome (dorm dust) and the top hits reported by MSE-based searches. (a) Distribution of OTUs in the common phylogeny tree between the query and the top hit from the full MSE reference database. Those abundant OTUs from the *Pseudomonas* genus are marked in the red box, and the shared subbranches of the query and the hits are indicated in blue. (b) The similarities between the query sample and each of the top 10 hits against the building reference samples are significantly lower than those between the query and each of the 10 hits against the entire database, as suggested by both *t* test (b) and PCoA (c). PC1 and PC2, principal components 1 and 2, respectively.

By comparing the query sample (i.e., dust from university dormitories) provided by Sun et al. (8) and the MSE top-hit samples, which are from mosquito tissues, we found that although abundant sequences of the two (query and the top-hit) samples are distributed among different OTUs (species) within the *Pseudomonas* genus, they are still very close in the common OTU-based phylogenetic tree (extracted from the Greengenes tree) (Fig. 1a), resulting in a high similarity of 0.916. To test whether this match is significant, we ranked this value in pairwise similarity calculation among all microbiomes (*n *=* *177,022) in MSE [in total, (*n* · *n* – 1)/2 = 15,668,305,731 times). The resulting *P* value of the permutation test is 0.0009, suggesting a highly significant match. This might have revealed potential interaction or transmission between mosquitos and dust, as these mosquitos were collected from residential properties and buildings (samples for generating 16S rRNA amplicon libraries were prepared by grinding one insect or a pool of individual insects [9]) (Table 1), or it might have highlighted communities that are distinct yet still dominated by microbes that are similar to one another when the overall picture of the bacterial tree is considered.

**TABLE 1:** Details for the top 10 hits for the query microbiome, dorm dust

MSE database ID of top 10 hit	Habitat	Similarity	Sampling location	Sampling date (yr/mo/day)	Reference
IDs from entire MSE database					
S_10815.C1OtvW34TOR2012	Mosquito tissue	0.91586	Toronto, Canada	2012/8/21	9
S_10815.NOjW34MSL2012	Mosquito tissue	0.91350	Toronto, Canada	2012/7/24	9
S_10815.3A081OjW32LAM2012	Mosquito tissue	0.91291	Toronto, Canada	2012/8/7	9
S_10815.Can2CxW32MSL2012	Mosquito tissue	0.91283	Toronto, Canada	2012/8/22	9
S_10815.O3AvW34TOR2012	Mosquito tissue	0.91260	Toronto, Canada	2012/6/12	9
S_10815.Y12A2AnpW31PEE2012	Mosquito tissue	0.91183	Toronto, Canada	2012/8/1	9
S_10815.C1AvW30TOR2012	Mosquito tissue	0.91134	Toronto, Canada	2012/7/24	9
S_10815.Can10AvW32MSL2012	Mosquito tissue	0.91097	Toronto, Canada	2012/8/15	9
S_10815.M1AvW32WEC2012	Mosquito tissue	0.91095	Toronto, Canada	2012/7/31	9
S_10815.B4AvW25TOR2013	Mosquito tissue	0.91088	Toronto, Canada	2013/6/18	9

IDs from “Building” subset of reference microbiomes in MSE database					
S_10172.815	Room surface dust	0.90388	Chicago, IL, USA	2017/5/24	10
S_10172.828	Nurse station surface dust	0.90063	Chicago, IL, USA	2017/5/24	10
S_1772.H23Cb	Kitchen cutting board	0.89745	Raleigh-Durham, NC, USA	2013/5/22	11
S_10172.286	Cold tap water	0.89666	Chicago, IL, USA	2017/5/24	10
S_10172.830	Nurse station surface dust	0.89300	Chicago, IL, USA	2017/5/24	10
S_SRR5574403	Kitchen dust	0.89109	Oakland, CA, USA	2017/5/17	12
S_10423.34E7LN0ZRJUQB	Carpet dust	0.88931	Toronto, Canada	2004/7/14	13
S_10172.10456	Cold tap water	0.88743	Chicago, IL, USA	2017/5/24	10
S_10172.8331	Glove	0.88592	Chicago, IL, USA	2017/5/24	10
S_10172.291	Room surface dust	0.88534	Chicago, IL, USA	2017/5/24	10

To test whether microbiomes from similar environments are more similar to each other than those from distinct environments, we next searched the query sample (which is dust collected inside a building) against all “building” samples in the reference database of MSE (a subset that includes 11,248 samples that were labeled as “building” from 35 studies). The similarities between the query and each of the top 10 hits (10–13) (Table 1) against the building reference samples are significantly lower than those between the query and each of the top 10 hits against the entire database (Fig. 1b) (*t* test *P* value = 2.75E–08). Findings from principal-component analysis (PCoA) support this conclusion, because the query sample is closer to the mosquito samples (i.e., to hits from the entire database) than to the building sample hits (i.e., hits from the building database) (Fig. 1c). These results suggest that microbiomes from similar environments can indeed be more different from each other than from certain samples from other environments that would intuitively be considered distinct.

In our current MSE implementation (1), the microbiome novelty score (MNS) is calculated based on the top hits against the whole reference database in MSE, rather than against only a subset of the reference microbiomes or those from a specific environment. We are grateful to Sun et al.’s suggestion of allowing the choice of reference databases when using MSE. In the upcoming release of MSE (http://mse.ac.cn), we plan to allow the selection of a specific environment or ecosystem as the reference database to search against, although we caution strongly that such restricted searches may lead to incorrect interpretation of results when the databases are not comprehensive.

Recently, amplicon sequence variant (ASV)-based approaches have been developed to improve the resolution of classifying 16S rRNA genes (14–16), but they require a unified sequencing platform and identical gene amplicon regions among the data sets. At present, the majority of historical microbiome samples were produced via a variety of platforms and amplicon regions; e.g., the V1-V3 and V3-V5 regions of 16S rRNA gene were sequenced via Roche 454 in the Human Microbiome Project (17), while the V4 region was sequenced via Illumina HiSeq and MiSeq in the Earth Microbiome Project (18). This reality limits the prospect of adopting the ASV scheme in MSE for searching against the current 16S rRNA-based microbiome data space. On the other hand, with the rapid accumulation of shotgun metagenomic data sets, we expect MSE to accommodate such data sets and eventually allow microbiome searches at the strain level, as Sun et al. have suggested.
